# Explaining Chinese Consumers’ Green Food Purchase Intentions during the COVID-19 Pandemic: An Extended Theory of Planned Behaviour

**DOI:** 10.3390/foods10061200

**Published:** 2021-05-26

**Authors:** Xin Qi, Angelika Ploeger

**Affiliations:** Specialized Partnerships in Sustainable Food Systems and Food Sovereignty, University of Kassel, 37213 Kassel, Germany; a.ploeger@uni-kassel.de

**Keywords:** green food, purchase intention, TPB, E-TPB, COVID-19, Chinese consumer

## Abstract

The outbreak of the COVID-19 pandemic has strongly influenced consumers’ habits and behaviours, creating a more sustainable and healthier era of consumption. Hence, there is a potential for further expanding the green food sector in China. The theory of planned behaviour (TPB) is one widely used framework to explain consumers’ food choices. Considering consumers’ internal norms, their perceptions of green food attributes, and the shifting consumer behaviour, our study has extended the TPB framework (E-TPB) by adding constructs of moral attitude, health consciousness, and the impact of COVID-19 (IOC). The results of structural equation modelling among 360 functional samples revealed that the E-TPB model has a superior explanatory and predictive power, compared with the original TPB model regarding Chinese consumers’ green food buying intentions in the current and post-pandemic periods. The path analysis demonstrated that attitude, perceived behavioural control, moral attitude, health consciousness, and IOC have significant positive effects on green food purchase intentions. However, the association between subjective norm and purchase intention varies within the TPB and E-TPB models, which showed a non-significant impact in E-TPB. These findings can generate more suitable managerial implications to promote green food consumption in China during the current and post-pandemic periods.

## 1. Introduction

In March 2020, the World Health Organization (WHO) announced the outbreak of the highly transmittable Coronavirus (COVID-19) as a pandemic [1], which is considered to be the third pandemic in the 21st century [2]. After one year’s development, there have been more than 138 million confirmed cases of COVID-19 globally and more than 751 million vaccine doses administered in April 2021 [1]. Although the situation is expected to improve in the next several years, the COVID-19 pandemic undoubtedly has widespread effects on society and consumers, hinting towards the dynamic changes in the market [3,4]. Some studies have found that there has been increased consumption of unhealthy food during the initial ‘lockdown’ period [5,6]. However, many studies have recently reported that consumers are increasingly concerned about the health and safety aspects of their food consumption and want to protect and strengthen their immune systems through their food diets [7,8,9,10]. In a 2021 survey from Accenture, an investigation involving more than 3000 consumers in 15 countries has shown that the pandemic is likely to create a more sustainable and healthier consumption era over the following 10 years [11]. This development can permanently alter consumer behaviours and cause lasting structural changes to products and industries [11]. Therefore, enterprises and marketers in the organic and green food sectors need to accelerate their business objectives and capabilities to match the shifting consumption patterns for their products and services during the current pandemic and post-pandemic periods.

Beginning in the 1990s, green food has been one of the most successful eco-labelling innovations in the Chinese food production industry [12]. Green food primarily refers to a full range of safe and premium edible agricultural products and related processed products, and are required to be grown in an ecologically sound environment, produced based on green food production standards, adopt the wholesome quality control, and granted the right to have a ‘green food’ certification [13]. There are two different standards for green food: Grade A and Grade AA. Grade A allows food producers to use limited chemical pesticides, chemical fertilisers, and other chemical inputs. Grade AA has stricter standards that exclude the guidelines mentioned above and are equivalent to Chinese organic food production standards [13]. After 30 years of development, Chinese green food production and consumption have experienced rapid growth and continued to expand in scale. According to official statistics in 2020, there have been 19,321 green food enterprises, 43,729 green-food-labelled products, and USD 66.52 billion of domestic sales, with a growing average of 9.2% annually [14]. Meanwhile, many Chinese families pay increasing attention to their health, quality lifestyles, environmental protection, and food security, and prefer to consume safe and green food products [15,16]. Despite undergoing exponential development and resulting in a booming trend, green food sales account for an extremely low percentage of the total food sales in China (i.e., less than 1%). Thus, there is an excellent prospect for the further expansion of green food consumption in China.

Since the Chinese green food industry faces an upward demand for sustainable consumption and growth potential, stakeholders in the green food sector need to understand the effective mechanisms of consumers’ green food purchase intentional behaviours during the current and post-pandemic periods in more detail. The theory of planned behaviour (TPB), proposed by Ajzen [17], is one of the most widely used theories predicting consumers’ purchase intentions of environmentally friendly food products [18,19]. According to Ajzen [17], the TPB is open for modification by incorporating new constructs or altering the path. Moreover, some nonnegligible limitations of applying TPB exist, and therefore, various refinements and extensions of TPB theory have been suggested to improve its effectiveness and applicability [20,21]. It is necessary to use multidisciplinary approaches for better understanding consumers’ food preferences and acceptances in different contexts and eating scenarios [22]. Consequently, certain important factors related to consumer behaviours during a pandemic should be investigated and validated to establish an updated green food purchase intention model.

Researchers and theorists have recurrently criticised the TPB for its insufficient consideration of other human behavioural constructs such as moral and ethical concerns [20]. Moral attitude is considered another salient behavioural factor in purchasing environmentally friendly food products since these behaviours are commonly perceived as pro-environmental actions [23]. Moreover, recent studies that investigated the influence of the COVID-19 pandemic on food consumption show that many consumers have been increasingly concerned about the health aspects [7,8,9]. Health concerns are considered as one of the significant drivers promoting consumers’ attitudes and intentions towards purchasing environmentally friendly food products [24]. Lastly, the emergence of the pandemic has strongly affected the global food systems, such as a collapse in a growing demand for global agri-food products, the severe disruptions of domestic and international food supply chains, the shortage of labour for food production enterprises, and the shifting consumption pattern [8,25,26]. Currently, no studies have established a green food purchase intention model with an integration of COVID-19 pandemic influences and incorporating salient factors among Chinese consumers. Therefore, it is necessary to adjust former models by integrating important and new factors that account for green food consumption and COVID-19 to understand consumers’ green food consumption better during a pandemic.

Hence, this paper aims to explore an appropriate model to explain and predict Chinese consumers’ green food purchase intentions during the current and post-pandemic periods. Based on the TPB, we have proposed an extended theory of planned behaviour (E-TPB) model and applied a structural equation modelling (SEM) approach to conduct model comparisons and examine the performance of each construct. Therefore, the outcome of this research can contribute to offering significant practical implications for researchers and marketers in the Chinese green food industry. The present study can generate new insight into the effects of the COVID-19 pandemic crisis on green food consumption. Moreover, marketers can use our findings to develop innovative marketing strategies to promote green food consumption in China further.

The remainder of this paper is organised as follows: Section 2 outlines the conceptual model developed for the present study and includes hypotheses to be examined; Section 3 explains the research methodology, which includes data collection, measurement, and data analysis; Section 4 displays descriptive statistics and SEM; Section 5 discusses of results and implications; Section 6 provides conclusions and includes research limitations and suggestions for future work.

## 2. Theoretical Framework and Development of Hypotheses

### 2.1. Theoretical Framework

**Figure 1:**
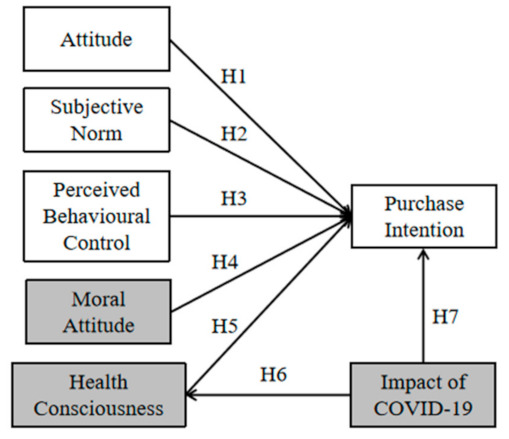
Research model: the white blocks are variables in the standard TPB model; the grey blocks and the white blocks are variables in the E-TPB model; H1, Hypothesis 1; H2, Hypothesis 2; H3, Hypothesis 3; H4, Hypothesis 4; H5, Hypothesis 5; H6, Hypothesis 6; H7, Hypothesis 7.

The present study has adopted TPB and proposed an E-TPB model by adding three constructs (i.e., moral norm, health consciousness, and the impact of COVID-19), to explain and predict Chinese consumers’ green food purchase intentions during the current and post-pandemic periods. The conceptual framework is represented in Figure 1.

### 2.2. Development of Hypotheses

#### 2.2.1. TPB

According to the TPB, there are three factors that collectively lead to the formation of an individual’s intentional behaviour: attitude, subjective norm, and perceived behavioural control [17].

- Attitude

Attitude assesses the extent to which people favourably or unfavourably evaluate the subject in question [17]. A subject can be a product, a person, or any other physical or intangible stimulus. There are substantial empirical studies on attitudes affecting consumers’ food choices towards environmentally friendly food products. Previous research emphasises a strong positive correlation between attitude and purchase intention towards organic food [27,28], green food [19], and sustainably sourced food [29]. Therefore, the present study introduces Hypothesis 1 (H1):

**Hypothesis** **1** **(H1).** *Chinese consumers’ attitudes towards green food products significantly influence their green food purchase intentions.*

- Subjective Norm

Subjective norm relates to perceived social influences or stresses to engage or disengage in a given behaviour [17]. Subjective norm also reveals the individuals’ beliefs about how their reference groups would view them if they perform a certain behaviour [28]. According to Scalco et al. [30], the most important social influences related to consumers’ environmentally friendly food purchases are from their families, friends, colleagues, and other reference groups. Previous research indicated that subjective norm is a positive driver of consumers’ behavioural intentions to indulge in their food choices [31,32]. However, other scholars [19,33] argue the efficacy of subjective norm in explaining consumer food choices and agree that more examinations into the role of the subjective norm are needed. Based on the aforementioned discussion, Hypothesis 2 (H2) is proposed as the following:

**Hypothesis** **2** **(H2).** *Subjective norm has a significant impact on Chinese consumers’ green food purchase intentions*.

- Perceived Behavioural Control (PBC)

PBC refers to an individual’s ability to control their behaviour independently [17]. Previous studies highlight PBC as a salient factor of intention during sustainable food consumption [34,35]. Studies from Yadav and Pathak [27] and Carfora et al. [36] have confirmed that the most significant impact of PBC on consumers’ buying intentions towards organic food products is mainly due to unavailability issues. Since some attributes of green and organic food products are similar, findings from consumer studies about organic food can serve as a reference for green food studies. Hence, Hypothesis 3 (H3) is presented as follows:

**Hypothesis** **3** **(H3).** *Perceived behavioural control is significantly related to Chinese consumers’ intentions to buy green food*.

#### 2.2.2. Incorporating Additional Constructs in the TPB

- Moral Attitude

Moral attitude refers to a person’s self-evaluation resulting from their expected compliance with their moral principles [37]. Moral attitude is considered to be a significant driver that impacts organic and green food consumption since consumers realise that their sustainable purchases can affect other people’s well-being. Thus, they perceive a sense of responsibility for their purchases and look for opportunities to fulfil them [29,38]. Studies from Dowd and Burke [29] and Yazdanpanah and Forouzani [39] have inserted moral norms into the TPB model. Both studies reported that when adding moral norms into the original model, there are significant increases in the fidelity and explanatory capability of the model. Hence, Hypothesis 4 (H4) is proposed based on the above discussion:

**Hypothesis** **4** **(H4).** *The moral attitude among Chinese consumers positively influences consumers’ intentions to purchase green food*.

- Health Consciousness

Health consciousness is defined as ‘the degree to which health concerns are integrated into a person’s daily activities’ [40], which reveals a person’s willingness to conduct health behaviours [41]. According to Paul and Rana [42], people who are more concerned about their health have frequent positive attitudes towards buying organic products since they are commonly considered as a healthier choice, compared to conventionally grown food varieties [24,43]. Many studies have found health consciousness as a significant motivator for consumer decisions towards environmentally friendly food products [44,45,46,47] and a crucial factor that strongly influences consumers’ willingness to pay for premium products [48]. Thus, Hypothesis 4 (H4) is proposed as follows:

**Hypothesis** **5** **(H5).** *Health consciousness among Chinese consumers positively influences consumers’ intentions towards buying green food*.

#### 2.2.3. Incorporating the Impact of COVID-19 (IOC) into the TPB Framework

The ongoing coronavirus pandemic crisis has caused a severe global health emergency and, consequently, has led to a shift in food systems as well as the way people purchase and consume their food [49]. The study from Meixner and Katt [3] has assessed the IOC on consumers’ perceptions about food safety issues. Their findings suggest that food safety concerns are becoming increasingly important due to COVID-19. Moreover, the latest studies [8,50] reported that the COVID-19 pandemic could lead people’s behaviours and lifestyles towards a sustainable and healthier direction. People tend to consume more environmentally friendly food products due to an increase in their food safety concerns [8,50]. With its specific attributes, green food meets the current demand. Therefore, we have added the IOC construct into the standard TPB model, and we aim to investigate its influence on consumers’ green food purchase intentions and health consciousness. Accordingly, Hypothesis 6 (H6) and Hypothesis 7 (H7) are proposed:

**Hypothesis** **6** **(H6).** *The impact of COVID-19 is significantly related to Chinese consumers’ health consciousness*.

**Hypothesis** **7** **(H7).** *The impact of COVID-19 is significantly related to Chinese consumers’ green food purchase intentions*.

## 3. Methodology

### 3.1. Data Collection

As part of the study, an online survey was applied to collect data and analyse the developed research framework. The geographic location used within the research was the Chinese mainland, a country containing the world’s largest food consumer group. Data were collected using a questionnaire survey platform (i.e., www.wenjuan.com (accessed on 24 May 2021)). After a brief pilot study involving 15 consumers, the initial questionnaire was adjusted and refined to improve comprehension and readability. The online questionnaire was distributed via WeChat, i.e., the most widely used mobile messaging application among Chinese people. Participants could answer the questionnaire by accessing the WeChat app with their smartphones. In addition, a certain number of ‘red packets’, which is an electronic monetary function in WeChat, were enabled to attract more consumers. The target group of this survey included consumers over the age of 20 due to the age category accounting for the majority of Chinese green consumers [51]. Therefore, the participant’s age was asked at the beginning of the online survey and was used for filtering. The survey would only continue if the age requirement was met. The survey was available to WeChat active users from 12 to 19 April 2021. A total of 398 questionnaires were returned and 38 of the 398 respondents were excluded due to the straight-line answer pattern and failure to complete the survey questions. A valid sample of 360 respondents (i.e., response rate = 90.4%) was used as a research dataset. According to Kline [52], there should be at least 10 cases per measurement as an acceptable sample size for conducting an experimental investigation. This study contains 19 measured items with a required minimum of 190 responses. Thereby, a total of 360 valid questionnaires was considered a valid sampling and sufficient for further data analysis. Table 1 provides an overview of the demographics of the samples.

### 3.2. Measurements

The scales used in this study were adopted from previous studies and modified to employ valid measurement instruments. This study considered the particularity of the Chinese language and culture. The contents were screened by two academic experts and back-translated by two local language experts to ensure accuracy and data integrity. Besides demographic questions, the other responses on the statements were obtained on a seven-point Likert scale (i.e., 1 for strongly disagree and 7 for strongly agree). The measurement items and their sources of adoption are shown in Table 2.

### 3.3. Data Method

SPSS Statistics version 24 and AMOS version 24 were applied to analyse the data and test the hypothetical associations between the constructs in the research model. Firstly, data were examined by descriptive statistics (i.e., means and standard deviations) to analyse the characteristics of participants and visualise the responses received. Secondly, confirmatory factor analysis (CFA) was conducted to measure the validity and reliability of measurement items within the proposed models. Next, we applied SEM to evaluate the model fit and hypothesis testing between TPB and E-TPB models. Finally, the indicators and hypothesis testing results of the two frameworks were compared and summarised based on data analysis results.

## 4. Results

### 4.1. Descriptive Statistics

**Figure 2:**
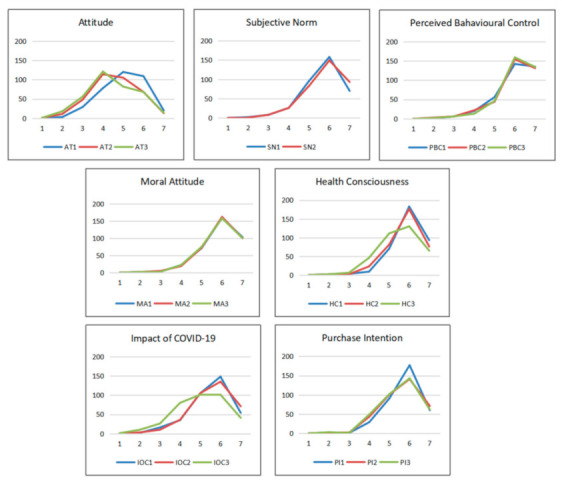
The overview of responses (*n* = 360): X-axis, seven-point scale (1 = strongly disagree, 2 = disagree, 3 = somewhat disagree, 4 = neither agree nor disagree, 5 = somewhat agree, 6 = agree, 7 = strongly agree); Y-axis, number of responses; AT, attitude; SN, subjective norm; PBC, perceived behavioural control; MA, moral attitude; HC, health consciousness; IOC, impact of COVID-19; PI, purchase intention.

In general, participants in this investigation expressed positive purchase intentions for green food (Mean = 5.645; SD = 0.872). For other determinants, participant responses were higher for PBC (Mean = 6.061; SD = 0.887), moral attitude (Mean = 5.909; SD = 0.827), health consciousness (Mean = 5.777; SD = 0.773), subjective norm (Mean = 5.750; SD = 0.910), and IOC (Mean = 5.381; SD = 1.007). Surprisingly, consumers’ answers for the attitude construct (Mean = 4.678; SD = 1.047) were neutral. Figure 2 presents the responses from all participants in this survey. Although some curves displayed slight fluctuations, most curves in general showed a homogeneous response pattern for all constructs. 

### 4.2. Measurement Model

Table 3 presents the results from the reliability and validity analysis of each measurement. All of the Cronbach’s α values were higher than the threshold of 0.7 [56], indicating that the questionnaire’s data have adequate reliability. In regard to convergent validity, all variables presented high composite reliability (CR), with scores ranging from 0.795 to 0.904, and were above the recommended standard of 0.6 [57]. In addition, the values of factors loading for all variables ranged from 0.719 to 0.944 and exceeded the acceptable value of 0.6 [58]. Additionally, the values of AVE (0.563 to 0.758) were above the acceptable limit of 0.5 [56]. Therefore, the convergent validity of the measurements was satisfied. Regarding the discriminant validity, the value of the square root of AVE was estimated for each variable and compared with its correlation value. As shown in Table 4, most construct pairs satisfied this requirement, except in one case (i.e., moral attitude and health consciousness). Therefore, the chi-square (∆χ^2^) difference test was performed regarding this lone problematic case. The chi-square difference test exhibited highly significant differences (∆χ^2^ = 182.139, *p* < 0.001). Therefore, discriminant validity was confirmed, which indicates that all variables used in the study were distinctively different.

### 4.3. Structural Model

The goodness-of-fit indices of the structural model are presented in Table 5. Regarding the original TPB framework, the structural TPB model demonstrated a good fit to the sample data, with χ^2^/df = 2.533, GFI = 0.956, TLI = 0.970, IFI = 0.980, CFI = 0.980, and RMSEA = 0.065. As for the proposed extended framework (i.e., E-TPB model), its goodness-of-fit indices (χ^2^/df = 2.870; GFI = 0.893; TLI = 0.938; IFI = 0.951; CFI = 0.950; RMSEA = 0.068) also showed satisfactory fit indices. Although the value of GFI (0.893) was slightly smaller than the suggested level (≥0.9), the structural model of E-TPB still can be accepted due to the good performance of other indices. Finally, the E-TPB model was compared with the standard TPB model. Our findings show that the E-TPB model has a better explanatory power (R^2^ = 0.68), in comparison to the original TPB (R^2^ = 0.49), for measuring Chinese consumers’ green food intentional purchases during the COVID-19 pandemic period. Notably, the extended model can explain 68% of the total variance in this study.

### 4.4. Hypotheses Testing

The path analysis results of the TPB and E-TPB models are presented in Table 6, including standardised parameter estimates, *t*-values, significance levels, and the results of each hypothesis. For the original variables of the TPB framework, the constructs of attitude (β = 0.395, *t* = 7.373, *p* < 0.001; β = 0.237, *t* = 4.806, *p* < 0.001, respectively) and PBC (β = 0.284, *t* = 4.153, *p* < 0.001; β = 0.122, *t* = 2.111, *p* < 0.05, respectively) had a significant impact on Chinese consumers’ green food purchase intentions in both TPB and E-TPB models. Therefore, hypotheses H1 and H3 are justified. However, the path analysis results of subjective norm (β = 0.188, *t* = 2.498, *p* < 0.05) were significant in the TPB model, but the results of subjective norm in the E-TPB model (β = −0.119, *t* = −1.478, *p* > 0.05) were nonsignificant and negatively related to consumers’ buying intentions of green food products. Thus, H2 is partially supported. Regarding the extended constructs in the E-TPB model, all the structural model results presented in Table 6 show the significant relationships between the three additional variables (i.e., moral norm, health consciousness, and IOC) and purchase intention, albeit with different levels of significance. Moral attitude (β = 0.318, *t* = 3.352, *p* < 0.001) and health consciousness (β = 0.154, *t* = 2.023, *p* < 0.05) affected consumers’ purchase intentions towards green food. Hence, H4 and H5 are supported. Regarding the role of IOC, there were significantly strong and positive associations between IOC and health consciousness (β = 0.600, *t* = 9.579, *p* < 0.001), as well as purchase intention (β = 0.315, *t* = 4.950, *p* < 0.001). Accordingly, H6 and H7 are supported.

## 5. Discussion

The present study explores Chinese consumers’ green food purchase intentions by developing and applying an extended model adapted to COVID-19 pandemic influences. A new E-TPB model was proposed by extending the original TPB model, incorporating three salient variables (i.e., moral norm, health consciousness, and IOC) into the framework. The results of our empirical investigation revealed better applicability in the E-TPB model than the TPB model and identified several key factors relating to Chinese consumers’ green food purchase intentions.

Regarding the original TPB constructs’ impact on green food intentional purchases, attitude and PBC were found to have a significant positive effect on Chinese consumers’ green food intentional behaviour. Consumers’ attitudes play a significant role in driving consumers’ intentions towards purchasing green food. When consumers have a positive attitude towards green food products, their intentions to buy green food increase. This result is consistent with former studies [19,27,28,29] involving environmentally friendly food products. Thus, green food enterprises need to make an effort to increase consumers’ positive perceptions and attitudes of green food products, such as highlighting the benefits of branded food and conducting promotional campaigns to increase consumers’ beliefs and knowledge. PBC is also an important aspect that is directly associated with consumers’ green food purchase intentions, which conforms with conclusions of previous studies [19,34,59]. Specifically, the present study applies a quantitative approach to validate the results of recent qualitative research [7] that the PBC is the influential antecedent of an intentional purchase of green food products during the COVID-19 pandemic. Hence, marketers in the Chinese green food sector should increase varieties and expand the supply channels of green food products. Online shopping is a trend for current food consumption in China, especially during the ongoing COVID-19 pandemic. Marketers in the green food industry can cooperate with a takeout platform (e.g., Eleme app) and retail podium (e.g., Freshhema app) to increase availability and convenience. Interestingly, the correlation of the association between consumers’ subjective norms and their green food buying intentions differed within the TPB and E-TPB models, which was significant in TPB but was negative and not significant in the E-TPB. A possible explanation of this incongruence is due to the unstable, poor predictive power of subjective norms, and variation in different contexts [60,61], especially regarding organic and green food purchases [32,39]. Qi and Ploeger [19] have substituted the subjective norm into the factors of face consciousness and group conformity when investigating Chinese consumers’ behaviour. The results from their study [19] showed that the replacement greatly improved the predictive power of explaining consumers’ intentional behaviour of green food products in the Chinese context.

In the extended model, the analysed results supported findings from studies involving the purchase of environmentally friendly food products [27,39] and demonstrated that consumers’ moral attitude towards green food is a significant positive driver of intentional purchases. In particular, moral attitude showed a significant effect since it resulted in a larger contribution to the explanatory power of the proposed E-TPB framework. Our findings suggest that more Chinese consumers feel it is a moral norm to buy green food products as their purchase intentions increase. Therefore, marketers can highlight concepts related to moral imperatives in their marketing strategies to influence consumers to gain positive feelings in purchasing green food. As expected, health consciousness emerges as one significant driver of green food purchase intention as well, which correlates with previous findings that the consumers’ health concern is one of the primary determinants influencing their environmentally friendly food behavioural intentions [62,63,64]. Thus, marketers in the Chinese green food industry should disseminate its health-related benefits and make it a primary objective while communicating with consumers. In regard to the IOC, our results indicated that there is a significant impact on consumers’ health consciousness and their purchase intentions during the pandemic. Our findings show that the pandemic has shifted an individual’s consumption pattern and structure, which is consistent with recent studies [4,49,50]. The pandemic has greatly increased an individual’s safety and health concerns, and people are increasingly focused on health benefits, which also supports findings from Meixner and Katt [3]. Thus, facing the rise in willingness and existing challenges, companies in the green food industry should quickly adjust their production, inspection, supply, and marketing strategies to better respond to the pandemic. For example, companies can provide information about virus and safety inspections with their product packages, increase online sales channels, prevent the upswing in prices, and strengthen promotional activities, especially in highlighting the benefits of green food products.

Finally, in terms of comparing the overall performance between the standard and extended frameworks, our results have validated the effectiveness of TPB and demonstrated the superior performance of E-TPB in regard to explaining and predicting Chinese consumers’ green food purchase intentions. Notably, the explanatory power difference between the original TPB model (R^2^ = 49%) and the E-TPB model (R^2^ = 68%) in predicting consumers’ intentions to buy green food products was higher than 19%. Hence, the E-TPB model is more appropriate for explaining and predicting Chinese consumers’ green food purchase intentions in the current and post-pandemic periods.

## 6. Conclusions

The present study has revealed that after the outbreak of COVID-19, the E-TPB model has exhibited better explanatory power in predicting Chinese consumers’ purchase intentions towards green food products when compared with the original TPB model. The findings from our investigation have reinforced existing evidence that factors including attitude, PBC, moral attitudes, health consciousness, and IOC have played significant roles in the intentional processes of buying green food during a pandemic crisis. In addition, our work is among the first attempt to explore the impacts of the COVID-19 pandemic on consumers’ green food purchase behaviour by distributing an online survey. Additionally, the above findings have potentially mapped a pathway to expand the green food market in China further. Our findings explored a newly developed model to gain a better understanding of how different and new factors affect consumers’ behavioural intentions towards green food purchases during a pandemic. In addition, certain limitations should be noted for further research. Firstly, our study has investigated an intentional stage, not the actual purchase behaviour of green food. Since the correlation between behavioural intentions and actual or observed behaviours is not always perfect [65], a further study can extend our framework to a final purchase behaviour phase to substantiate current research findings. Secondly, we used an online survey platform to collect data. This approach may result in sample bias because consumers without internet access were not included in our samples. The education levels among our respondents could have been over-represented in our investigation. Thereby, our findings could not be considered representative of the whole population. Accordingly, a future investigation should enlarge the sample size and investigate more diverse populations from different backgrounds. Thirdly, recent studies [66,67,68] have reported that there is an increasing need from consumers to receive food information services. Therefore, further research should consider the important role of food information and incorporate it within our framework and then apply the framework to green food research. Finally, since the pandemic is constantly changing and there exist large uncertainties, further research can update and modify our proposed model according to shifting consumer behaviours and consumption patterns.

## Figures and Tables

**Table 1:** Demographics of samples (*n* = 360).

Demographics Variables		Frequency	Percent (%)
Gender	Male	166	46.1
	Female	194	53.9
Age	20–30	124	34.4
	31–40	87	24.2
	41–50	83	23.0
	51–60	42	11.7
	>60	24	6.7
Marital Status	Married with a child or children	149	41.4
	Married	80	22.2
	Single	115	32.0
	Other	16	4.4
Education	Junior school or below	57	15.9
	High school or technical secondary school	124	34.4
	University or above	179	49.7
Monthly Income (RMB)	<4500	98	27.2
	4500–9000	174	48.3
	>9000	88	24.5

**Table 2:** Measurement of constructs.

Constructs	Items	Measurement Items	Adopted From
Purchase Intention (PI)	PI1	I prefer to choose green food products if they are available for purchase.	Yazdanpanah and Forouzani [39]
	PI2	In the near future, I will try to buy green food.	
Attitude (AT)	AT1	I think purchasing green food is a good concept.	Wang et al. [53]
	AT2	I believe buying green food is pleasant.	
	AT3	I believe buying green food is of importance.	
Subjective Norm (SN)	SN1	Most people I value believe I should purchase green food.	Han et al. [54]
	SN2	Most people I value will purchase green food rather than non-green food.	
Perceived Behavioural Control (PBC)	PBC1	If I want to, I can easily buy green food.	Han et al. [54]
	PBC2	I have all resources for buying green food.	
	PBC3	Buying green food is entirely up to me.	
Moral Attitude (MA)	MA1	If I purchase green food rather than non-green food, it feels like a personal contribution to something better.	Arvola et al. [55]
	MA2	If I purchase green food rather than non-green food, it feels like I’m doing the morally right thing.	
	MA3	If I purchase green food rather than non-green food, I feel like I’m being a better person.	
Health Consciousness (HC)	HC1	I chose food carefully to ensure good health.	Yadav and Pathak [27]
	HC2	I consider myself a health-conscious consumer.	
	HC3	I often think about health-related issues.	
Impact of COVID-19 (IOC)	IOC1	I perceive the COVID-19 pandemic has influenced me personally.	Meixner and Katt [3]
	IOC2	I perceive the COVID-19 pandemic will shift my consumption pattern.	
	IOC3	I perceive the COVID-19 pandemic will change society.	

**Table 3:** Reliability and validity analysis.

Constructs	Factor Loadings		CR		SMC		AVE		Cronbach’s α	√AVE
	TPB	E-TPB	TPB	E-TPB	TPB	E-TPB	TPB	E-TPB		
PI			0.904	0.901			0.758	0.752	0.902	0.867
PI1	0.840	0.838			0.706	0.703				
PI2	0.870	0.866			0.757	0.751				
PI3	0.901	0.897			0.813	0.805				
AT			0.893	0.894			0.738	0.740	0.888	0.859
AT1	0.733	0.743			0.538	0.553				
AT2	0.944	0.942			0.891	0.888				
AT3	0.887	0.883			0.786	0.780				
PBC			0.900	0.900			0.750	0.750	0.899	0.866
PBC1	0.864	0.864			0.747	0.746				
PBC2	0.886	0.887			0.785	0.787				
PBC3	0.847	0.846			0.717	0.716				
SN			0.830	0.834			0.710	0.716	0.830	0.843
SN1	0.826	0.789			0.682	0.623				
SN2	0.859	0.900			0.739	0.810				
MA				0.841				0.640	0.849	0.800
MA1		0.845				0.714				
MA2		0.830				0.688				
MA3		0.719				0.516				
HC				0.795				0.563	0.792	0.750
HC1		0.778				0.605				
HC2		0.737				0.544				
HC3		0.736				0.541				
IOC				0.891				0.731	0.887	0.855
IOC1		0.905				0.818				
IOC2		0.844				0.712				
IOC3		0.814				0.662				

Note: PI, purchase intention; AT, attitude; PBC, perceived behavioural control; SN, subjective norm; MA, moral attitude; HC, health consciousness; IOC, impact of COVID-19; CR, composite reliability; SMC, squared multiple correlation; AVE, average variance extracted; √AVE, square root of average variance extracted.

**Table 4:** Correlation matrix for discriminant validity.

	SN	IOC	HC	MA	PBC	AT	PI
SN	0.843						
IOC	0.458	0.855					
HC	0.599	0.569	***0.750***				
MA	0.755	0.529	***0.771***	0.800			
PBC	0.670	0.432	0.561	0.630	0.866		
AT	0.478	0.527	0.421	0.440	0.376	0.859	
PI	0.550	0.693	0.686	0.700	0.550	0.594	0.867

Note: The diagonal elements represent the square root of AVE; off-diagonal elements show the correlations between constructs; values in italics boldface indicate that values for the shared variance are larger than the square root of AVE values; SN, subjective norm; IOC, impact of COVID-19; HC, health consciousness; MA, moral attitude; PBC, perceived behavioural control; AT, attitude; PI, purchase intention.

**Table 5:** Goodness-of-fit indices and explanatory power of two models.

Models	χ^2^/df	GFI	TLI	IFI	CFI	RMSEA	R^2^
Thresholds	>1 and <5 *	≥0.9 *	≥0.9 *	≥0.9 *	≥0.9 *	≤0.08 *	
TPB	2.533	0.956	0.970	0.980	0.980	0.065	0.49
E-TPB	2.870	0.893	0.938	0.951	0.950	0.068	0.68

Note: * Source from Bagozzi and Yi [57]; GFI, goodness-of-fit index; NFI, normative fit index; TLI, Tucker–Lewis index; CFI, comparative fit index; IFI, incremental fit index; RMSEA, root mean square error approximation.

**Table 6:** Hypotheses test results.

Hypothesised Path	Standardised Estimate		*t*-Value		Result
	TPB	E-TPB	TPB	E-TPB	
H1: AT → PI	0.395	0.237	7.373 ***	4.806 ***	Support
H2: SN → PI	0.188	−0.119	2.498 *	−1.478	Partly support
H3: PBC → PI	0.284	0.122	4.153 ***	2.111 *	Support
H4: MA → PI		0.318		3.352 ***	Support
H5: HC → PI		0.154		2.023 *	Support
H6: IOC → HC		0.600		9.579 ***	Support
H7: IOC → PI		0.315		4.950 ***	Support

Note: *** *p* < 0.001; ** *p* < 0.01; * *p* < 0.05; AT, attitude; PI, purchase intention; SN, subjective norm; PBC, perceived behavioural control; MA, moral attitude; HC, health consciousness; IOC, impact of COVID-19.

## Data Availability

The datasets generated and/or analysed during the current study are not publicly available due data are not public but are available from the corresponding author on reasonable request.
